# Meniscal Extrusion Correlates with Symptom Severity in Knee Osteoarthritis: An Ultrasound and Magnetic Resonance Imaging Analysis of 100 Patients

**DOI:** 10.3390/jcm13247716

**Published:** 2024-12-18

**Authors:** Fabio Tortorella, Angelo Boffa, Alessandro Di Martino, Luca Andriolo, Giancarlo Facchini, Maddalena Di Carlo, Marco Miceli, Stefano Zaffagnini, Giuseppe Filardo

**Affiliations:** 1 Applied and Translational Research (ATR) Center, IRCCS Istituto Ortopedico Rizzoli, 40136 Bologna, Italy; fabio.tortorella@ior.it; 2 Department of Shoulder and Elbow Unit, IRCCS Istituto Ortopedico Rizzoli, 40136 Bologna, Italy; 3 Clinica Ortopedica e Traumatologica 2, IRCCS Istituto Ortopedico Rizzoli, 40136 Bologna, Italy; angelo.boffa@ior.it (A.B.); luca.andriolo@ior.it (L.A.); stefano.zaffagnini@ior.it (S.Z.); 4 Diagnostic and Interventional Radiology Unit, IRCCS Istituto Ortopedico Rizzoli, 40136 Bologna, Italy; giancarlo.facchini@ior.it (G.F.); marco.miceli@ior.it (M.M.); maddalena.dicarlo@ior.it (M.D.C.); 5 Faculty of Biomedical Sciences, Università Della Svizzera Italiana, CH-6900 Lugano, Switzerland; ortho@gfilardo.com

**Keywords:** extrusion, meniscus, ultrasounds, magnetic resonance, osteoarthritis, cartilage

## Abstract

**Purpose**: The aim of this study was to investigate how meniscal extrusion, assessed either with ultrasounds or magnetic resonance (MR), correlates with clinical symptoms in knee osteoarthritis (OA). **Methods:** One hundred patients with symptomatic knee OA were enrolled (60.3 ± 9.7 years). Patients underwent MR evaluation and ultrasound analyses (clinostatic and orthostatic positions). Patients were clinically evaluated through IKDC, KOOS, WOMAC, VAS, and Tegner scores. Correlation analyses were performed between meniscal extrusion extent and clinical scores. Lower (<4 mm) and higher extrusion (≥4 mm) groups were also compared. **Results:** The identification of low/high extrusion was 56/44 (MR) and 45/55 (ultrasounds) for patients with medial meniscus and 72/28 (MR) and 57/43 (ultrasounds) for patients with lateral meniscus. Meniscal extrusion correlated with symptoms (*p* < 0.05) with worse clinical findings in patients with higher extrusion, particularly for the lateral meniscus. For the medial meniscus, more differences were found between lower and higher extrusion groups with ultrasounds than MR, especially in the orthostatic position, while for the lateral meniscus, similar trends were documented with both methods. **Conclusions:** Extrusion of both menisci correlates with knee OA symptoms, with a stronger correlation for the lateral meniscus. Ultrasounds performed in the standing position identify more patients with meniscal extrusion and correlate better than MR with clinical findings.

## 1. Introduction

Knee osteoarthritis (OA) is one of the most common orthopedic joint diseases, with over 300 million people affected worldwide [1]. The pathological processes are due to a complex interaction of genetic, biomechanical, and metabolic factors leading to joint inflammation and degeneration [2]. Knee OA is characterized by symptoms including pain, swelling, and functional disability that reduce the quality of life and lead to a substantial social and economic burden [3,4]. The underlying mechanisms of OA-related pain are complex and multifactorial, including structural damage such as cartilage degeneration and osteophyte formation, synovial inflammation, subchondral bone alterations, and meniscal pathology [5]. There are still gaps in our understanding of the connections between the different structural damages affecting knee OA and the development of symptoms. While previous evidence suggests the influence of synovitis or subchondral bone involvement in symptom generation, knowledge about the influence of the meniscus remains limited. Hence, understanding these factors is crucial for improving management strategies and patient outcomes.

Meniscal extrusion has recently gained increasing attention among the structural abnormalities observed in knee OA [6]. This phenomenon is defined as the displacement of the meniscus beyond the tibial plateau ≥3 mm and is a consequence of the interactions of joint tissue degeneration and mechanical stresses leading to the OA disease [7]. Growing evidence showed that meniscal extrusion is correlated with greater knee OA severity and faster disease progression, as it alters joint biomechanics and worsens load distribution, accelerating damages to both cartilage and subchondral bone [8,9,10,11]. Magnetic resonance (MR) imaging remains the gold standard for assessing meniscal extrusion, although it is performed in the supine position and, therefore, meniscus behavior under load-bearing cannot be evaluated. Recent studies underlined the importance of evaluating meniscal extrusion by ultrasounds, due to its advantage of performing meniscal extrusion measurements in both supine and standing positions [12,13,14]. Moreover, US assessment is also more broadly available, timesaving, portable, and cheaper compared to MR imaging [15,16,17]. Despite the large interest in meniscal extrusion in the knee OA process, its relationship with clinical symptoms, such as pain and functional impairment, remains unclear. Previous studies suggest a relationship between the presence of meniscal extrusion and knee OA symptoms [15,16]; however, it is not clear if the entity of the extrusion and the evaluation method can influence this relationship.

The aim of this study was to investigate how meniscal extrusion, assessed either with ultrasounds or MR correlates with clinical symptoms in patients affected by knee OA. The hypothesis is that greater symptomatology could be correlated with a higher degree of meniscal extrusion and that evaluation using ultrasound could be more accurate than MR imaging.

## 2. Materials and Methods

### 2.1. Study Design and Patient Selection

This study was approved by the hospital Ethics Committee of the IRCCS Istituto Ortopedico Rizzoli, Italy (Prot. Nr 0001673). Patients were enrolled by orthopedic physicians between March 2021 and April 2023 in a research outpatient clinic specialized in patients with knee OA. Informed consent was obtained from each patient for study participation. Male and female patients aged between 18 and 80 years with signs and symptoms of knee OA and radiographic evidence of knee OA (Kellgren–Lawrence grade 1–4) were enrolled in this study. The following exclusion criteria were used: patients unable to express consent, patients who performed knee surgery in the previous 12 months, patients suffering from malignant tumor or rheumatic diseases, patients with a history of a sub-total or total meniscectomy of the affected knee, and patients with axial deviation > 5°. One hundred consecutive patients with symptomatic knee OA were enrolled according to inclusion and exclusion criteria. Patients’ characteristics are reported in Table 1.

### 2.2. Imaging Analysis of Meniscal Extrusion

**Figure 1:**
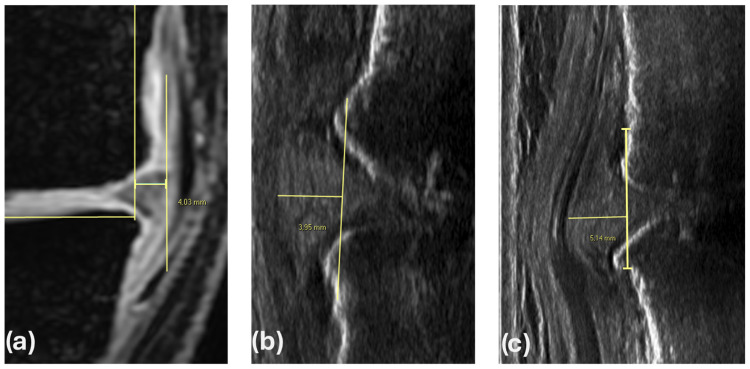
Measurement of meniscal extrusion in a male patient (51 years) using magnetic resonance (**a**), ultrasounds in the clinostatic position (**b**) and ultrasounds in the orthostatic position (**c**).

All patients underwent imaging evaluations of the affected knee, including MR and ultrasound analyses (Figure 1). MR was performed independently by the patients in different settings, while the measurement was performed by the investigators consistently in the same way for all patients. Meniscal extrusion on MR was measured on the central coronal slice where the medial tibial spine was most visible. The reference point for measuring the extrusion was the osteochondral limit of the tibial plateau, taking into account and excluding potential osteophytes. A line connecting the medial and lateral osteochondral junctions (tibial width) was used as a baseline. A perpendicular line was drawn at the borders, and meniscal extrusion was measured medially and laterally relative to this baseline. Ultrasound evaluation was conducted with the knee fully extended in the clinostatic (clino-US) and then in the orthostatic (ortho-US) position. A high-frequency linear probe at 5–12 MHz was used for meniscal extrusion evaluation. For the medial meniscus, longitudinal ultrasound imaging was obtained by aligning the probe parallel to the medial collateral ligament. Meniscal displacement was measured as the distance from the outermost meniscal edge to a line connecting the femoral and tibial cortical bones, as per Kawaguchi et al. [17]. For the lateral meniscus, the protocol by Winkler et al. [18] was followed, using landmarks such as the fibular head and the lateral collateral ligament. Quantitative extrusion measurements were performed in millimeters by two experienced radiologists in consensus.

### 2.3. Clinical Evaluation

All patients were clinically evaluated through the International Knee Documentation Committee (IKDC) objective and subjective score, the Knee injury and Osteoarthritis Outcome Score (KOOS) subscales, the Western Ontario and McMaster Universities Arthritis Index (WOMAC), the visual analogical scale (VAS) for pain, and the Tegner score for the sport/activity level. The clinical scores are reported in Table 2. To evaluate the influence of meniscal extrusion on clinical symptoms, correlation analyses were performed between the extent of meniscal extrusion and the clinical scores assessed. Moreover, to further investigate the influence of meniscal extrusion on the clinical status, the included patients were categorized in two groups using a 4 mm cut-off of meniscal extrusion for both the medial and lateral menisci: lower extrusion group (<4 mm of extrusion) and higher extrusion group (≥4 mm of extrusion) [19].

### 2.4. Statystical Analysis

All continuous data were expressed in terms of the mean and the standard deviation of the mean and range. The Shapiro–Wilk test was performed to test the normality of continuous variables. The Levene test was used to assess the homoscedasticity of the data. The ANOVA test was performed to assess the between groups differences in continuous, normally distributed and homoscedastic data, the Mann–Whitney nonparametric test was used otherwise. The Spearman rank correlation was used to assess correlations between numerical data; the Kendall tau rank correlation was used for ordinal data. The Pearson Chi square evaluation using the exact test was performed to investigate relationships between grouping variables. For all tests >0.05 was considered significant. All statistical analysis was performed using SPSS v.19.0 (IBM Corp., Armonk, NY, USA)

## 3. Results

### 3.1. Meniscal Extrusion and Clinical Scores

**Figure 2:**
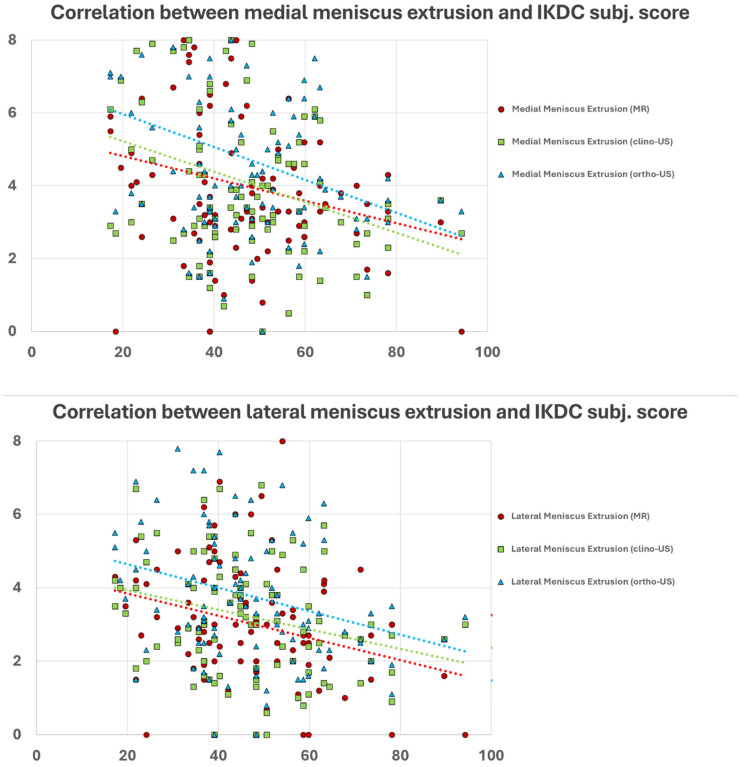
Negative correlations between meniscal extrusion and the IKDC subjective score. *X*-axis, IKDC Subjective Score; *Y*-axis, meniscal extrusion magnitude in millimeters. Dashed lines indicate trend lines for each imaging assessment (red, MR; green, clino-US; blue, ortho-US).

The quantitative analysis of the medial meniscus documented an average extrusion of 4.0 ± 2.1 mm at the MR evaluation, 4.1 ± 2.1 mm at the clino-US evaluation, and 4.8 ± 2.1 mm at the ortho-US evaluation. The extrusion of the lateral meniscus was 3.0 ± 1.6 mm at the MR evaluation, 3.2 ± 1.7 mm at the clino-US evaluation, and 3.8 ± 1.9 mm at the ortho-US evaluation. Overall, the entity of meniscal extrusion negatively correlated with the clinical symptoms of the patients, with worse clinical findings in patients with higher meniscal extrusion. This correlation was documented for both menisci and for all three imaging evaluations (Figure 2), as reported in Table 3. Further investigations have been performed separately for both medial and lateral meniscus based on the classification of patients into lower extrusion and higher extrusion groups.

### 3.2. Medial Meniscus: Lower vs. Higher Extrusion

Based on the MR measurements of the medial meniscus extrusion, the lower extrusion group was composed of 56 patients (mean age 57.4 ± 9.7 years), while the higher extrusion group was composed of 44 patients (mean age: 64.0 ± 8.3 years). The two groups were different in terms of age (*p* = 0.001) and Kellgren–Lawrence grade (*p* = 0.001). Regarding clinical scores, the higher extrusion group presented a worse clinical status compared to the lower extrusion group in terms of IKDC subjective score (41.7 ± 15.1 vs. 50.5 ± 15.1, *p* = 0.006) and KOOS QoL (36.6 ± 18.6 vs. 44.8 ± 19.3, *p* = 0.031). Based on the clino-US evaluation of the medial meniscus extrusion, the lower extrusion group was composed of 56 patients (mean age 56.4 ± 9.3 years), while the higher extrusion group was composed of 44 patients (mean age: 65.4 ± 7.6 years); based on the ortho-US evaluation of the medial meniscus extrusion, the lower extrusion group was composed of 45 patients (mean age 55.4 ± 9.6 years), while the higher extrusion group was composed of 55 patients (mean age: 64.4 ± 7.7 years). The two groups were different in terms of age (*p* < 0.0005), BMI (*p* = 0.003), and Kellgren–Lawrence grade (*p* < 0.0005). Overall, a higher number of statistically significant differences were found between the lower extrusion group and the higher extrusion group with the ultrasound evaluation compared with MR, as reported in detail in Table 4.

### 3.3. Lateral Meniscus: Lower vs. Higher Extrusion

Based on MR measurements of the lateral meniscus extrusion, the lower extrusion group was composed of 72 patients (mean age 59.4 ± 9.3 years), while the higher extrusion group was composed of 28 patients (mean age 62.8 ± 10.3 years). The two groups were different in terms of Kellgren–Lawrence grade (*p* = 0.003). Regarding clinical scores, the higher extrusion group presented a worse clinical status compared to the lower extrusion group in terms of IKDC subjective score (40.3 ± 13.6 vs. 49.1 ± 15.8, *p* = 0.016), KOOS Pain (57.2 ± 15.5 vs. 68.8 ± 16.2, *p* = 0.002), KOOS Symptom (57.7 ± 19.8 vs. 69.3 ± 16.3, *p* = 0.007), KOOS ADL (65.1 ± 17.1 vs. 74.8 ± 17.1, *p* = 0.007), and all WOMAC subscales (*p* < 0.05). Based on the clino-US evaluation of the lateral meniscus extrusion, the lower extrusion group was composed of 66 patients (mean age 58.6 ± 10.2 years), while the higher extrusion group was composed of 34 patients (mean age: 63.8 ± 7.6 years); based on the ortho-US evaluation of the medial meniscus extrusion, the lower extrusion group was composed of 57 patients (mean age 58.0 ± 10.6 years), while the higher extrusion group was composed of 43 patients (mean age: 63.4 ± 7.3 years). The two groups were different in terms of age (*p* = 0.009), BMI (*p* = 0.01), and Kellgren–Lawrence grade (*p* = 0.002). Overall, similar trends were documented between the lower extrusion group and the higher extrusion group with the ultrasound evaluation compared with MR, as reported in detail in Table 5.

## 4. Discussion

The main finding of this study is that the extrusion of both medial and lateral menisci correlates with the symptoms experienced by knee OA patients in terms of pain, stiffness, and functional activities, with a stronger correlation for the lateral meniscus. The evaluation with ultrasounds in the standing position is able to identify more patients with meniscal extrusion and correlates more than MR with the clinical findings.

This study, analyzing a large cohort of 100 patients affected by symptomatic knee OA, highlighted that meniscal extrusion significantly affects the severity of the symptoms. Meniscal extrusion alters joint biomechanics, exacerbating cartilage degeneration, and subchondral bone damage, which are key contributors to pain development and functional impairment in knee OA patients [5,9]. The findings of this study demonstrate that these changes translate into a worse clinical outcome, including increased pain, stiffness, and reduced functionality. This underlines the pivotal role of meniscal pathology in knee OA disease, being not a simple structural abnormality but rather a critical factor determining OA-related symptoms. A previous analysis from the Osteoarthritis Initiative investigated the relationship between meniscal pathology evaluated at MR imaging and the severity of symptoms in knee OA [20], although patients were categorized by the authors as having meniscal either morphological deformity or extrusion and, together, did not report a significant association with knee pain. Unlike this analysis, the current study specifically focused on meniscal extrusion, which led to the identification of a significant correlation with the symptoms. The impact of meniscal extrusion on symptoms was confirmed by stratifying patients based on meniscal extrusion severity. Patients with greater extrusion (≥4 mm) reported significantly worse clinical outcomes, which is consistent with previous research demonstrating the direct correlation between higher meniscal extrusion and more advanced OA severity [6,9].

The study also demonstrated that lateral meniscal extrusion has a stronger impact on symptom severity compared to medial extrusion, presenting a higher number of correlations with a worse clinical status. This finding is in line with previous biomechanical studies suggesting that the lateral compartment experiences higher dynamic loads, making the knee more susceptible to biomechanical imbalance and symptomatic deterioration when the lateral meniscus is extruded [21,22]. The stronger correlation between extrusion and symptoms for the lateral meniscus could be highlighted already with MR, despite its limitations in terms of underestimating the meniscal extrusion versus the ultrasound evaluation [12]. For the medial meniscus, the more accurate evaluation using ultrasound was required instead to fully identify statistically significant correlations between extrusion and symptoms. In fact, the assessment of the influence of medial meniscus extrusion on symptoms found twice as many correlations when evaluated with ultrasound compared to MR imaging. This highlights the importance of assessing medial meniscus extrusion using ultrasound in a weight-bearing position.

Ultrasound evaluation, especially when performed in the standing position, is emerging as a valuable tool for quantifying meniscal extrusion. Compared to MR imaging, ultrasound offers real-time, functional imaging, capturing the effects of weight-bearing on meniscal extrusion. Recent literature highlighted its utility, with a recent study demonstrating that ultrasound evaluation outperforms MR imaging in quantifying meniscal extrusion in patients with knee OA [12]. Moreover, the highest values of meniscal extrusion have been documented using ultrasound in the standing position compared to the supine position, underlining the importance of the weight-bearing assessment [12]. A previous study analyzed 85 patients affected by knee OA in the supine position with ultrasounds to investigate possible correlations between ultrasonographic findings and clinical scores [23]. The authors reported that the presence of medial meniscal extrusion was correlated with clinical scores, while no significant correlations were found for the lateral meniscal extrusion. Unlike that study, the current study investigated the influence of the level of meniscal extrusion, finding a significant influence of both medial and lateral meniscal extrusion on clinical outcomes. Moreover, this study also analyzed the OA knees in the standing position, confirming the importance of ultrasound evaluation in weight-bearing, with higher values of meniscal extrusion detected by ultrasound evaluation with the patient in a standing position, and a stronger correlation between the entity of meniscal extrusion and the severity of the symptoms. This protocol may possibly be applied also in animal studies in order to better understand the role of the meniscus in OA in vivo models and the response to treatments [24]. Moreover, the correct identification of meniscal extrusion may also guide the development of surgical procedures aiming at reducing the extrusion and improving the symptoms.

This study presents some limitations. While this study included a high number of knee OA patients and a three-level imaging analysis, differences in patient characteristics, such as age, BMI, and Kellgren–Lawrence grade between the lower and higher extrusion groups, could introduce confounding effects. Furthermore, the observational design precluded establishing the influence of meniscal extrusion on the evolution of symptoms severity. Future longitudinal studies should investigate the evolution of symptomatology over time in correlation with meniscal extrusion. Despite these limitations, this study is of clinical importance, since understanding the correlation between meniscal extrusion and clinical findings can have significant implications for OA management. Recent studies demonstrated that meniscal extrusion greater than 2.2 and 2.8 mm is correlated with unsatisfactory Lysholm and IKDC subjective scores, respectively, in patients who underwent isolated medial meniscectomy [25]. Other studies, instead, emphasize a correlation with the dissatisfaction rate in clinical scores when meniscal extrusion measured on MRI is greater than 3 mm [26,27]. Therefore, an accurate assessment of meniscal extrusion, especially with ultrasound in weight-bearing conditions, can favor a better understanding of a key determinant of OA symptomatology, also facilitating early detection and intervention.

## 5. Conclusions

The extrusion of both medial and lateral menisci correlates with the symptoms experienced by knee OA patients, with a stronger correlation for the lateral meniscus. The evaluation with ultrasounds in a standing position is able to identify more patients with meniscal extrusion and correlates more than MR with the clinical findings. This knowledge can guide future research and personalize therapeutic strategies in clinical practice for a more tailored management of patients affected by symptomatic knee OA.

## Figures and Tables

**Table 1:** Baseline characteristics of the included patients.

Gender (Male/Female)	65/35
Age, y (mean ± SD)	60.3 ± 9.7
BMI, kg/m^2^ (mean ± SD)	25.8 ± 3.7
Symptoms duration, mo (mean ± SD)	80.5 ± 73.9
Kellgren–Lawrence grade	Grade 1:2 Grade 2:55 Grade 3:36 Grade 4:7

BMI, body mass index; mo, months; SD, standard deviation; y, years.

**Table 2:** Clinical characteristics of the included patients.

IKDC Subjective	46.6 ± 15.6
IKDC Objective	2.4 ± 0.8
KOOS Pain	65.5 ± 16.7
KOOS Symptoms	66.0 ± 18.0
KOOS ADL	72.1 ± 17.6
KOOS Sport/rec	46.4 ± 20.4
KOOS QOL	41.2 ± 19.3
WOMAC Pain	5.4 ± 3.5
WOMAC Stiffness	2.9 ± 2.7
WOMAC Function	19.8 ± 11.1
WOMAC Total	28.1 ± 15.7
VAS pain	5.2 ± 2.3
Tegner score	2.4 ± 1.3

IKDC, International Knee Documentation Committee; KOOS, Knee injury and Osteoarthritis Outcome Score; VAS, visual analog scale; WOMAC, Western Ontario and McMaster Universities Arthritis Index.

**Table 3:** Correlation between meniscal extrusion and clinical scores.

Score	MME (MR)	MME (Clino-US)	MME (Ortho-US)	LME (MR)	LME (Clino-US)	LME (Ortho-US)
IKDC subjective	Rho: −0.212 *p* = 0.034 *	Rho: −0.260 *p* = 0.009 *	Rho: −0.291 *p* = 0.003 *	Rho: −0.286 *p* = 0.004 *	Rho: −0.260 *p* = 0.009 *	Rho: −0.273 *p* = 0.006 *
IKDC objective	Tau: 0.204 *p* = 0.009 *	Tau: 0.211 *p* = 0.007 *	Tau: 0.245 *p* = 0.002 *	Tau: 0.135 *p* = 0.087	Tau: 0.154 *p* = 0.050	Tau: 0.163 *p* = 0.038 *
KOOS pain	Rho: −0.154 *p* = 0.125	Rho: −0.206 *p* = 0.040 *	Rho: −0.235 *p* = 0.019 *	Rho: −0.329 *p* = 0.001 *	Rho: −0.292 *p* = 0.003 *	Rho: −0.279 *p* = 0.005 *
KOOS symptoms	Rho: −0.059 *p* = 0.559	Rho: −0.107 *p* = 0.290	Rho: −0.045 *p* = 0.660	Rho: −0.399 *p* < 0.0005 *	Rho: −0.288 *p* = 0.004 *	Rho: −0.261 *p* = 0.009 *
KOOS ADL	Rho: −0.162 *p* = 0.108	Rho: −0.176 *p* = 0.080	Rho: −0.235 *p* = 0.019 *	Rho: −0.375 *p* < 0.0005 *	Rho: −0.253 *p* = 0.011 *	Rho: −0.246 *p* = 0.014 *
KOOS Sport/rec	Rho: −0.143 *p* = 0.157	Rho: −0.164 *p* = 0.104	Rho: −0.198 *p* = 0.049 *	Rho: −0.340 *p* = 0.001 *	Rho: −0.228 *p* = 0.023 *	Rho: −0.213 *p* = 0.033 *
KOOS QOL	Rho: −0.211 *p* = 0.036 *	Rho: −0.196 *p* = 0.051	Rho: −0.257 *p* = 0.010 *	Rho: −0.351 *p* < 0.0005 *	Rho: −0.301 *p* = 0.002 *	Rho: −0.217 *p* = 0.030 *
WOMAC total	Rho: 0.176 *p* = 0.081	Rho: 0.180 *p* = 0.073	Rho: 0.237 *p* = 0.017 *	Rho: 0.385 *p* < 0.0005 *	Rho: 0.231 *p* = 0.021 *	Rho: 0.247 *p* = 0.013 *
WOMAC pain	Rho: 0.177 *p* = 0.079	Rho: 0.197 *p* = 0.050	Rho: 0.248 *p* = 0.013 *	Rho: 0.303 *p* = 0.002 *	Rho: 0.235 *p* = 0.019 *	Rho: 0.243 *p* = 0.015 *
WOMAC stiffness	Rho: 0.140 *p* = 0.165	Rho: 0.166 *p* = 0.098	Rho: 0.208 *p* = 0.038 *	Rho: 0.397 *p* < 0.0005 *	Rho: 0.237 *p* = 0.018 *	Rho: 0.265 *p* = 0.008 *
WOMAC function	Rho: 0.163 *p* = 0.105	Rho: 0.165 *p* = 0.101	Rho: 0.223 *p* = 0.026 *	Rho: 0.367 *p* < 0.0005 *	Rho: 0.212 *p* = 0.035 *	Rho: 0.221 *p* = 0.027 *
VAS pain	Rho: 0.072 *p* = 0.480	Rho: 0.063 *p* = 0.533	Rho: 0.126 *p* = 0.212	Rho: −0.002 *p* = 0.982	Rho: 0.049 *p* = 0.627	Rho: 0.102 *p* = 0.311
Tegner score	Rho: −0.119 *p* = 0.238	Rho: −0.212 *p* = 0.035 *	Rho: −0.186 *p* = 0.064	Rho: −0.187 *p* = 0.062	Rho: −0.188 *p* = 0.062	Rho: −0.133 *p* = 0.185

Clino-US, Ultrasonographic evaluation in Clinostatic position; IKDC, International Knee Documentation Committee; KOOS, Knee injury and Osteoarthritis Outcome Score; LME, Lateral meniscal extrusion; MME, Medial Meniscal Extrusion; MR, Magnetic Resonance; Ortho-US, Ultrasonographic evaluation in Orthostatic position; VAS, visual analog scale; WOMAC, Western Ontario and McMaster Universities Arthritis Index. * *p* < 0.05.

**Table 4:** Differences in clinical scores between the lower and higher extrusion groups in relation to the medial meniscal extrusion.

Scores	Medial Meniscal Extrusion								
	MR			Clino-US			Ortho-US		
	<4 mm (56 pts)	≥4 mm (44 pts)	*p* Value	<4 mm (56 pts)	≥4 mm (44 pts)	*p* Value	<4 mm (45 pts)	≥4 mm (55 pts)	*p* Value
IKDC subjective	50.5 ± 15.1	41.7 ± 15.1	0.006 *	49.8 ± 16.8	42.5 ± 15.6	0.042 *	50.9 ± 17.2	43.1 ± 13.4	0.035 *
IKDC objective	2.2 ± 0.7	2.6 ± 0.8	n.s.	2.2 ± 0.7	2.6 ± 0.7	0.034 *	2.1± 0.7	2.6 ± 0.7	0.001 *
KOOS pain	68.5 ± 14.7	61.7 ± 18.5	n.s.	68.0 ± 14.9	62.3 ± 18.5	n.s.	68.6 ± 15.1	63.0 ± 17.7	n.s.
KOOS symptoms	67.2 ± 18.3	64.6 ± 17.7	n.s.	67.7 ± 17.6	63.9 ± 18.4	n.s.	67.3 ± 18.4	65.0 ± 17.7	n.s.
KOOS ADL	75.2 ± 15.1	68.1 ± 19.8	n.s.	74.5 ± 15.4	68.9 ± 19.8	n.s.	75.5 ± 15.7	69.2 ± 18.7	n.s.
KOOS Sport/rec	49.5 ± 21.3	42.5 ± 18.8	n.s.	48.8 ± 21.7	43.3 ± 18.3	n.s.	50.9 ± 21.8	42.6 ± 18.5	0.043 *
KOOS QOL	44.8 ± 19.3	36.6 ± 18.6	0.031 *	43.6 ± 19.5	38.1 ± 18.9	n.s.	45.3 ± 20.1	37.8 ± 18.2	n.s.
WOMAC total	25.1 ± 13.5	31.9 ± 17.7	n.s.	26.2 ± 13.9	30.6 ± 17.7	n.s.	25.5 ± 14.1	30.3 ± 16.8	n.s.
WOMAC pain	4.8 ± 3.1	6.2 ± 3.8	n.s.	5.0 ± 3.2	5.9 ± 3.8	n.s.	4.9 ± 3.3	5.9 ± 3.7	n.s.
WOMAC stiffness	2.4 ± 1.5	3.5 ± 3.7	n.s.	2.5 ± 1.5	3.4 ± 3.7	n.s.	2.4 ± 1.5	3.3 ± 3.4	n.s.
WOMAC function	17.9 ± 9.8	22.3 ± 12.2	n.s.	18.7 ± 10.0	21.3 ± 12.2	n.s.	18.2 ± 10.2	21.2 ± 11.6	n.s.
VAS pain	5.0 ± 2.2	5.5 ± 2.4	n.s.	5.1 ± 2.3	5.4 ± 2.3	n.s.	5.0 ± 2.3	5.4 ± 2.3	n.s.
Tegner score	2.5 ± 1.4	2.1 ± 1.2	n.s.	2.6 ± 1.4	2.0 ± 1.0	0.026 *	2.6 ± 1.4	2.1 ± 1.1	0.049 *

Clino-US, Ultrasonographic evaluation in Clinostatic position; IKDC, International Knee Documentation Committee; KOOS, Knee injury and Osteoarthritis Outcome Score; MR, Magnetic Resonance; n.s., not significant; Ortho-US, Ultrasonographic evaluation in Orthostatic position; VAS, visual analog scale; WOMAC, Western Ontario and McMaster Universities Arthritis Index. * *p* < 0.05.

**Table 5:** Differences in clinical scores between the lower and higher extrusion groups in relation to the lateral meniscal extrusion.

Scores	Lateral Meniscal Extrusion								
	MR			Clino-US			Ortho-US		
	<4 mm (72 pts)	≥4 mm (28 pts)	*p* Value	<4 mm (66 pts)	≥4 mm (34 pts)	*p* Value	<4 mm (57 pts)	≥4 mm (43 pts)	*p* Value
IKDC subjective	49.1 ± 15.8	40.3 ± 15.6	0.016 *	49.9 ± 16.3	40.2 ± 12.6	0.006 *	51.1 ± 16.6	43.1 ± 13.4	0.003 *
IKDC objective	2.3 ± 0.8	2.4 ± 0.7	n.s.	2.3 ± 0.7	2.5 ± 0.8	n.s.	2.3± 0.7	2.5 ± 0.8	n.s.
WOMAC total	25.1 ± 14.9	35.8 ± 15.4	0.001 *	25.0 ± 14.6	34.2 ± 16.4	0.006 *	24.3 ± 14.8	33.2 ± 15.7	0.006 *
WOMAC pain	4.8 ± 3.3	7.0 ± 3.7	0.006 *	4.6 ± 3.2	6.9 ± 3.7	0.004 *	4.5 ± 3.1	6.6 ± 3.7	0.006 *
WOMAC stiffness	2.4 ± 1.5	4.0 ± 4.5	0.011 *	2.4 ± 1.5	3.8 ± 4.1	0.028 *	2.3 ± 1.5	3.6 ± 3.7	0.011 *
WOMAC function	17.9 ± 11.0	24.9 ± 9.7	0.003 *	17.9 ± 10.7	23.5 ± 11.0	0.015 *	17.4 ± 10.9	23.0 ± 10.6	0.013 *
KOOS pain	68.8 ± 16.2	57.2 ± 15.5	0.002 *	70.2 ± 15.2	56.5 ± 16.0	<0.0005 *	70.6 ± 15.6	58.7 ± 15.8	0.001 *
KOOS symptoms	69.3 ± 16.3	57.7 ± 19.8	0.007 *	70.1 ± 16.2	58.0 ± 18.7	0.002 *	70.1 ± 16.4	60.6 ± 18.7	0.014 *
KOOS ADL	74.8 ± 17.1	65.1 ± 17.1	0.007 *	75.6 ± 16.1	65.2 ± 18.5	0.007 *	75.9 ± 16.7	67.0 ± 17.7	0.012 *
KOOS Sport/rec	48.0 ± 22.2	42.1 ± 14.1	n.s.	48.8 ± 22.0	41.6 ± 16.0	n.s.	50.2 ± 22.7	41.3 ± 15.7	0.019
KOOS QOL	43.5 ± 19.1	35.0 ± 18.9	n.s.	44.5 ± 19.6	34.7 ± 17.3	0.024 *	45.3 ± 19.6	35.6 ± 17.8	0.018 *
VAS pain	5.2 ± 2.3	5.3 ± 2.3	n.s.	5.1 ± 2.3	5.4 ± 2.3	n.s.	4.9 ± 2.3	5.7 ± 2.2	n.s.
Tegner	2.5 ± 1.3	2.1 ± 1.2	n.s.	2.6 ± 1.3	1.8 ± 1.0	0.002 *	2.6 ± 1.4	2.1 ± 1.2	0.045 *

Clino-US, Ultrasonographic evaluation in Clinostatic position; IKDC, International Knee Documentation Committee; KOOS, Knee injury and Osteoarthritis Outcome Score; MR, Magnetic Resonance; Ortho-US, Ultrasonographic evaluation in Orthostatic position; WOMAC, Western Ontario and McMaster Universities Arthritis Index;. VAS, visual analog scale. * *p* < 0.05.

## Data Availability

Data are contained within the article.
